# Piloting Temperature-Driven Variability in Emergency Diagnostic Accuracy Using a Leading Large Language Model

**DOI:** 10.7759/cureus.94476

**Published:** 2025-10-13

**Authors:** Philip C Jarrett, Jared Hill, Marshall Howell, Kristen Grabow Moore, Joby J Thoppil, Laura Vargas Ortiz, Samuel Parnell, Daniel M Courtney, Samuel McDonald, Deborah Diercks, Andrew Jamieson, Dazhe J Cao

**Affiliations:** 1 Emergency Medicine, University of Texas Southwestern Medical Center, Dallas, USA; 2 Emergency Medicine, Emory University, Atlanta, USA; 3 Biostatistics, University of Texas Southwestern Medical Center, Dallas, USA

**Keywords:** artificial intelligence in medicine, clinical decision support, clinical informatics, diagnostic accuracy, emergency medicine

## Abstract

Background

Large language models (LLMs) use a parameter known as temperature to control the stochasticity of output sampling during text outputs, which may have implications for clinical diagnostic tasks. In this study, we aimed to determine the impact of the temperature parameter on GPT-4o’s diagnostic accuracy when evaluating emergency medicine cases and assess the effect on diagnostic divergence across iterations.

Methodology

We conducted a simulation-based diagnostic accuracy study using four challenging emergency medicine cases adapted from the Foundations of Emergency Medicine curriculum. Each case was submitted to GPT-4o 250 times at five temperature settings (0.0, 0.25, 0.50, 0.75, 1.0), both with and without physical examination findings, yielding 10,000 total outputs. Each output contained exactly three differential diagnoses with one leading diagnosis to limit the inflation of diagnostic accuracy by larger, unprioritized lists of remotely possible diagnoses. Diagnostic accuracy was assessed by comparing outputs against predetermined gold-standard diagnoses. Mixed-effects models evaluated the relationship between temperature and diagnostic accuracy, while a sensitivity analysis excluded physical examination data. Diagnostic divergence, defined as the number of unique diagnoses generated across iterations, was explored within cases as a representation of internal consistency.

Results

At temperature 0.0, GPT-4o achieved 100% leading diagnosis accuracy across all cases with physical examination data. As the temperature increased, the accuracy declined systematically to 89.4% at the temperature setting of 1.0. Mixed-effects models demonstrated that temperature was inversely associated with correct leading diagnosis (β = -4.16, odds ratio (OR) = 0.02, 95% confidence interval (CI) = 0.01-0.03, p < 0.001) and with inclusion of the gold-standard diagnosis anywhere in the differential (β = -3.75, OR = 0.02, 95% CI = 0.01-0.05, p < 0.001). Diagnostic divergence increased from an average of 4.5 unique diagnoses at temperature 0.0 to 26.25 at temperature 1.0 (483% increase). Case sensitivity varied significantly, with ascending cholangitis showing the greatest temperature sensitivity (accuracy dropping from 100% to 70.4%), while carbon monoxide poisoning maintained 100% accuracy across all settings. Sensitivity analysis evaluating the impact of physical examination on diagnostic accuracy revealed case-specific effects. While the diagnostic accuracies of ascending cholangitis and myxedema coma were heavily affected by the exclusion of physical examination data, the carbon monoxide and cryptococcal meningitis cases were minimally changed, if at all.

Conclusions

Increasing the GPT-4o temperature parameter systematically introduced diagnostic inaccuracy across four emergency medicine vignettes. Lower temperature settings led to improved diagnostic accuracy and consistency across case iterations, which may make them preferable for clinical applications requiring high reliability. Transparent reporting of temperature settings is essential for reproducible clinical artificial intelligence research.

## Introduction

Large language models (LLMs) such as OpenAI’s GPT-4o have demonstrated significant medical capabilities, with studies suggesting diagnostic accuracy comparable to physicians [1-6]. These models are being applied to clinical decision support, particularly in high-stakes environments such as emergency medicine (EM) [7-12]. LLMs are also integral to increasingly popular ambient dictation solutions that formulate clinical notes on behalf of physicians [13]. As their clinical applications expand, ensuring the reliability and reproducibility of LLM-generated guidance has become a central concern.

LLM performance is inherently non-deterministic; responses vary with repetitions of a given task and can be influenced by various parameters [14-18]. Among these, temperature is a key setting that controls the randomness of a model’s output [14-18]. A low temperature (e.g., 0.0 or 0.2) consistently leads to focused, predictable outputs, whereas a high temperature (e.g., 0.8 or 1.0) increases randomness by allowing the model to sample less likely responses. This can yield more diverse outputs but also increases the risk of inappropriate or implausible answers in clinical contexts.

Temperature may therefore impact the reliability of LLM-generated medical guidance [17,18]. When an LLM proposes differential diagnoses in a clinical note, a low temperature may favor the most probable diagnoses while overlooking rarer possibilities. In contrast, a high temperature may encourage broader diagnostic exploration at the expense of introducing “noise” or improbable suggestions. Selecting an appropriate temperature thus represents a trade-off between diagnostic consistency and creative exploration, a balance that has direct implications for the safety and trustworthiness of LLM-assisted medical reasoning.

Despite its importance, temperature settings are underreported in LLM clinical research, limiting reproducibility [1-3,16-19]. Much of the existing literature on diagnostic performance challenges models with rare conditions, misrepresenting the data distributions encountered in daily practice [1-3]. Moreover, the default temperature settings in commercial chatbots are often unknown to the user, further eroding trust in outputs intended for clinical decision support [14]. There is a clear need to systematically evaluate how temperature affects LLM performance on medical tasks, particularly in realistic scenarios where clinicians must reason with limited early-encounter data, such as initial history of present illness, vital signs, and brief physical findings. The primary objective of this study was to determine the impact of the temperature parameter on GPT-4o’s diagnostic accuracy when evaluating high-risk EM cases based on early clinical information. Secondary objectives included assessing the effect of temperature on diagnostic divergence and the consistency of model outputs across a range of temperature settings.

## Materials and methods

Study design and case selection

**Figure 1:**
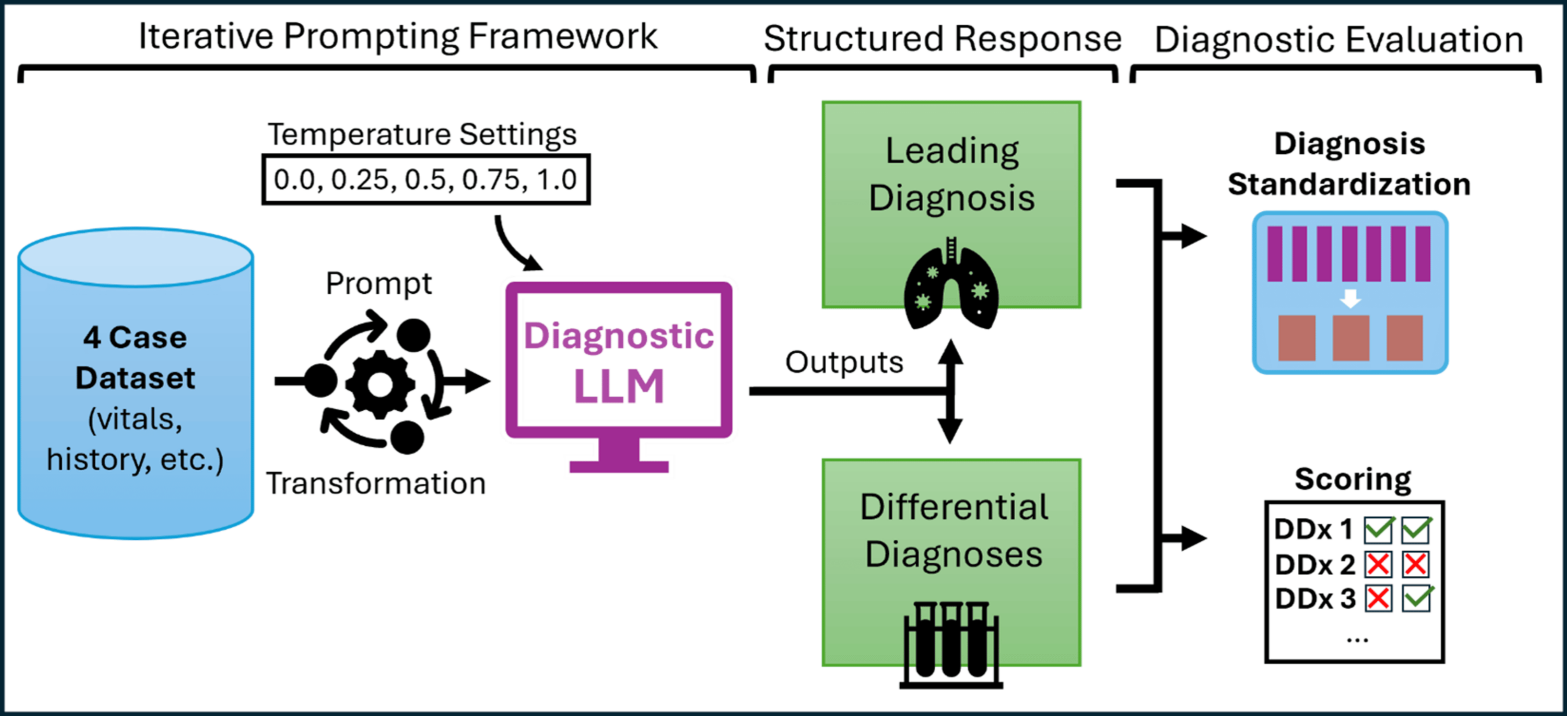
Study flow diagram. Study flowchart demonstrating the diagnostic evaluation pipeline using a large language model (LLM) and output scoring. Structured cases from Foundations of Emergency Medicine were adapted to represent early-encounter patient information. A standardized prompt specified the diagnostic reasoning task and output structure. The diagnostic LLM (GPT-4o) returned structured responses. Diagnostic lists (DDx) were scored for accuracy while unique diagnoses were standardized to assess diagnostic breadth.

We conducted a diagnostic accuracy pilot study using GPT-4o (Figure 1). Four challenging EM cases were adapted from the Foundations of Emergency Medicine (FoEM) curriculum, a widely adopted training resource protected by login, minimizing the likelihood of inclusion in GPT-4o’s training data [20,21]. In this pilot, we limited the evaluation to four cases to permit empiric high-volume sampling for each case-temperature pair. Case selection was guided by several criteria, as determined by editors of the FoEM curriculum. Each case must lead to a single unambiguous diagnosis. The clinical information must represent a cross-sectional snapshot of an early patient encounter (chief complaint, vitals, HPI, past medical/surgical history, and physical examination). The cases must reflect diagnoses spanning a range of organ systems. Final case selection was driven by consensus of two active FoEM editors (MH, KG) and the lead author (PJ) upon review of the available cases that met all inclusion criteria.

The diagnoses associated with these cases, as determined by FoEM editors at the time of case creation, served as the gold standard for assessing diagnostic accuracy (Table 1). The selected cases were myxedema coma (Endocrinology), ascending cholangitis (Infectious Disease/Gastroenterology), carbon monoxide Poisoning (Toxicology), and cryptococcal meningitis (Neurology/Infectious Disease).

**Table 1:** Selected cases. Details of the four cases selected from the Foundations of Emergency Medicine curriculum to evaluate GPT-4o’s diagnostic accuracy. HPI: history of present illness; PMH: past medical history; PSH: past surgical history; HR: heart rate in beats per minute; BP: blood pressure in mmHg; RR: respiratory rate in breaths per minute; Sat: oxygen saturation expressed as a percentage; T: body temperature in Celsius; HEENT: head, eyes, ears, nose, throat; ROS: review of systems

Diagnosis	Complaint/Vitals	HPI	PMH/PSH	Physical examination
Myxedema coma	Altered mental status – HR, 58; BP, 90/66; RR, 9; Sat, 96; T, 35.5	A 62-year-old female who lives alone was found lying in bed by her neighbor after she hadn’t been seen for the past 4 days. The neighbor reports that she is unsure of her medical history but knows that she sees multiple doctors. Further history and ROS are limited by the patient’s altered mental status	PMH: unknown; PSH: unknown	General: wakes to voice but immediately falls back asleep; HEENT: normal; chest: nontender; lungs: normal; heart: bradycardic, no murmurs; abdomen: normal; rectal: normal; extremities: 2+ non-pitting edema of the legs; back: normal; neuro: oriented to self only, follows simple commands, falls asleep easily, otherwise non-focal; skin: dry skin
Ascending cholangitis	Altered mental status – HR, 110; BP, 100/58; RR, 20; Sat, 93; T, 35.8	This is an 81-year-old female who presents with fatigue, malaise, and vomiting overnight. The nurse at the patient’s nursing facility noticed that she was more confused than usual and unable to get out of bed. She had an additional episode of nonbloody, nonbilious emesis right before EMS arrived. At baseline, she is a pleasant, ambulatory woman who is typically oriented to self but not time or place. ROS is otherwise negative	PMH: hypertension; PSH: hysterectomy	General: cachectic, fatigued; HEENT: dry mucous membranes, mild scleral icterus; neck: normal; heart: tachycardic, no murmurs, 1+ distal pulses; lungs: normal breath sounds bilaterally; abdomen: soft, mildly distended, patient grimaces with palpation of the right upper quadrant; rectal: normal; extremities: normal; Back: normal; neuro: mumbling incoherently, oriented x 0, eyes open, moving all extremities and localizing to pain; skin: jaundice, cool skin, 3 cm pressure ulcer (stage 1) to sacrum; lymph: normal
Carbon monoxide poisoning	Headache – HR, 110; BP, 90/50; RR, 24; Sat, 94; T 37.0	A 45-year-old male presents with headache, dizziness, and nausea. He reports he has been wearing extra layers to stay warm this winter. He reports that the headache is mild and diffuse, throbbing with slight nausea. He denies any neck stiffness, fever, or head injuries. ROS is otherwise negative	PMH: none; PSH: none	General: awake and alert, malodorous, no acute distress; HEENT: normal; neck: normal; chest: nontender; heart: tachycardic, otherwise normal; lungs: tachypneic, otherwise normal; abdomen: normal; extremities: normal; back: normal; neuro: slightly ataxic gait, otherwise normal; skin: normal
Cryptococcal meningitis	Headache – HR, 106; BP, 97/52; RR, 16; Sat, 98; T 37.7	A 27-year-old male presents with a frontal headache and intermittent chills for the past 3 days. This headache is unlike prior headaches, came on gradually, and is now 8/10 in intensity. He also reports myalgias and vomiting that started after the headache. He also notes chronic diarrhea for several months with associated weight loss. ROS is otherwise negative	PMH: none; PSH: none	General: thin male, mildly confused on interview, appears uncomfortable and fatigued; HEENT: dry mucous membranes, no thrush, otherwise normal; neck: supple; heart: tachycardic, otherwise normal; lungs: normal; abdomen: normal; genital: normal; extremities: normal; back: normal; neuro: awake and alert, oriented to person and place, but disoriented to year, no focal neuro deficits; skin: warm and clammy, otherwise normal

Because physical examination data are often unavailable during early clinical encounters, we conducted a sensitivity analysis excluding physical examination to assess the impact on diagnostic performance.

Language model selection

GPT-4o (model version gpt-4o-2024-05-13 via the OpenAI Application Programming Interface) was chosen as the LLM for this study due to its widespread availability, cost-effectiveness, and strong performance in early medical benchmarks [14]. Other output decoding parameters aside from temperature were controlled at their default values to avoid confounding, and the random seed could not be controlled due to restrictions imposed by the model provider. Because model responses were restricted to a structured JavaScript Object Notation (JSON) output schema and GPT-4o does not use inference tokens, max_tokens was not specified.

Iterative sampling and prompting

Temperature parameters typically span a range of 0.0 (low randomness) to 2.0 (high randomness). In the absence of prior literature on appropriate sample sizes to measure the empiric distribution of diagnostic outputs for LLMs, each of the four cases was iterated 250 times at five distinct temperature settings (0.0, 0.25, 0.50, 0.75, and 1.0), both with and without physical examination findings. Temperatures above 1.0 were excluded because preliminary testing indicated impaired adherence to prompt instructions at higher values. Iterative prompting across all case-temperature pairs resulted in 10,000 total outputs.

All prompting followed a structured format designed to simulate consultation with an artificial intelligence (AI) diagnostic assistant. The full system prompt is provided in Table 2. To standardize evaluation, the model was restricted to producing exactly three differential diagnoses per case, including one leading diagnosis. This constraint served several purposes. First, limiting output length reduced the risk of inflated diagnostic accuracy that can occur when evaluating correctness within long, unranked lists of conditions. This issue has been previously noted as a pattern of overtesting by LLMs, maximizing sensitivity at the cost of specificity [22]. Second, it better reflects real-world diagnostic decision-making, where clinicians apply heuristics to prioritize clinical possibilities when determining workup and treatment. Third, a three-item limit helped isolate the model’s prioritization behavior, forcing it to make judgment calls about what diagnoses warrant inclusion under constraint. This structure also enabled assessment of diagnostic divergence across iterations by observing variability in top-ranked differentials across multiple runs.

**Table 2:** Diagnostic assistant system prompt and output schema for GPT-4o output generation. The individualized case details were adjusted for each of the four cases. When physical examination data were excluded during sensitivity analysis, the examination details were replaced by the statement: “unavailable at this time.”

System prompt
You are a Large Language Model assisting with emergency department diagnostic evaluations. You will be provided with patient data (chief complaint, vitals, history of present illness, past medical/surgical history, and physical exam) collected during triage evaluation of the patient. Your task is to produce exactly three differential diagnoses for the most likely underlying condition, including a leading diagnosis and two additional differential diagnoses. Diagnoses should be as specific as possible. Do not use imprecise diagnostic terms. Do not use terms such as “possible” or “probable” in the diagnoses. Each diagnosis should be a single condition without qualifiers. Chief Complaint: {{Case-specific complaint}} Vitals: {{Case-specific vitals}} History of Present Illness: {{Case-specific history of present illness}} Past Medical/Surgical History: {{Case-specific past medical/surgical history}} Physical Examination: {{Case-specific exam findings}} You must respond ONLY with JSON (JavaScript Object Notation) in the following format, with no additional text or markdown: {“differential_diagnoses”: [“diagnosis1”, “diagnosis2”, “diagnosis3”], “diagnostic_tests”: [“test1”, “test2”, “test3”]} Requirements: - The response must be pure JSON with no other text - differential_diagnoses must be an array with exactly 3 strings - diagnostic_tests must be an array with at least one string - Do not include any explanations or markdown formatting

Each prompt began with a standardized header, which included instructions about the diagnostic task, expected output formatting, and clinical information about a single case (chief complaint, vital signs, history of present illness, past medical history, and, if applicable, physical examination findings). In the sensitivity analysis excluding physical examination data, this section was replaced with the phrase “unavailable at this time.” Prompts were designed to be stateless and were submitted in parallel, ensuring that the model had no memory of prior case iterations. Additionally, internet search and external reference capabilities were disabled during all interactions, requiring GPT-4o to rely exclusively on its embedded knowledge to generate diagnostic responses.

Outcome measures

**Figure 2:**
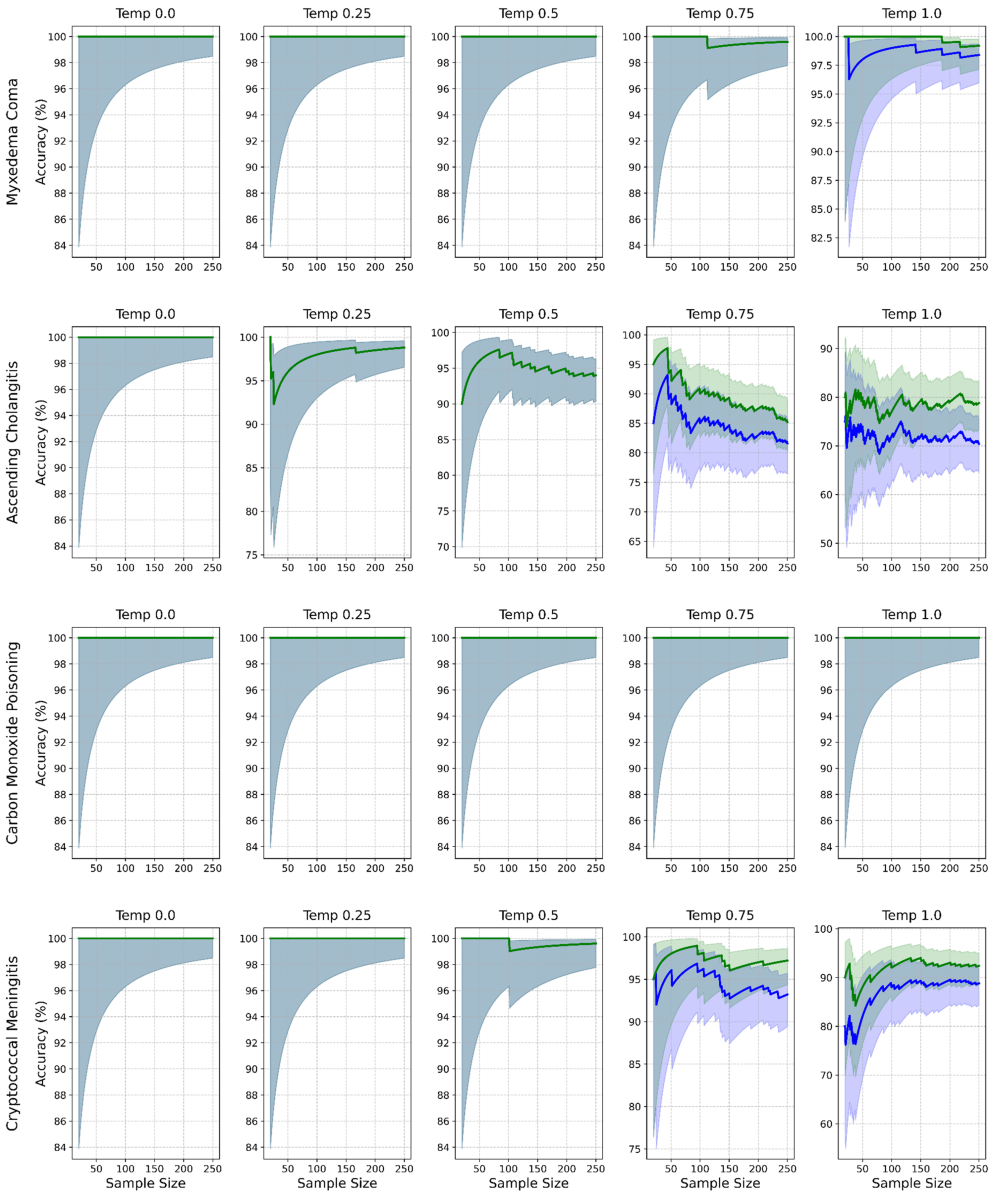
Diagnostic accuracy with physical examination data. Diagnostic accuracy by case (rows) and temperature (columns) with physical examination data. Lines show accuracy for leading (blue) and differential (green) diagnoses. Shaded purple and green areas represent the 95% confidence interval (CI) for the leading and differential diagnoses, respectively. Blue-gray areas represent overlap in 95% CI.

**Figure 3:**
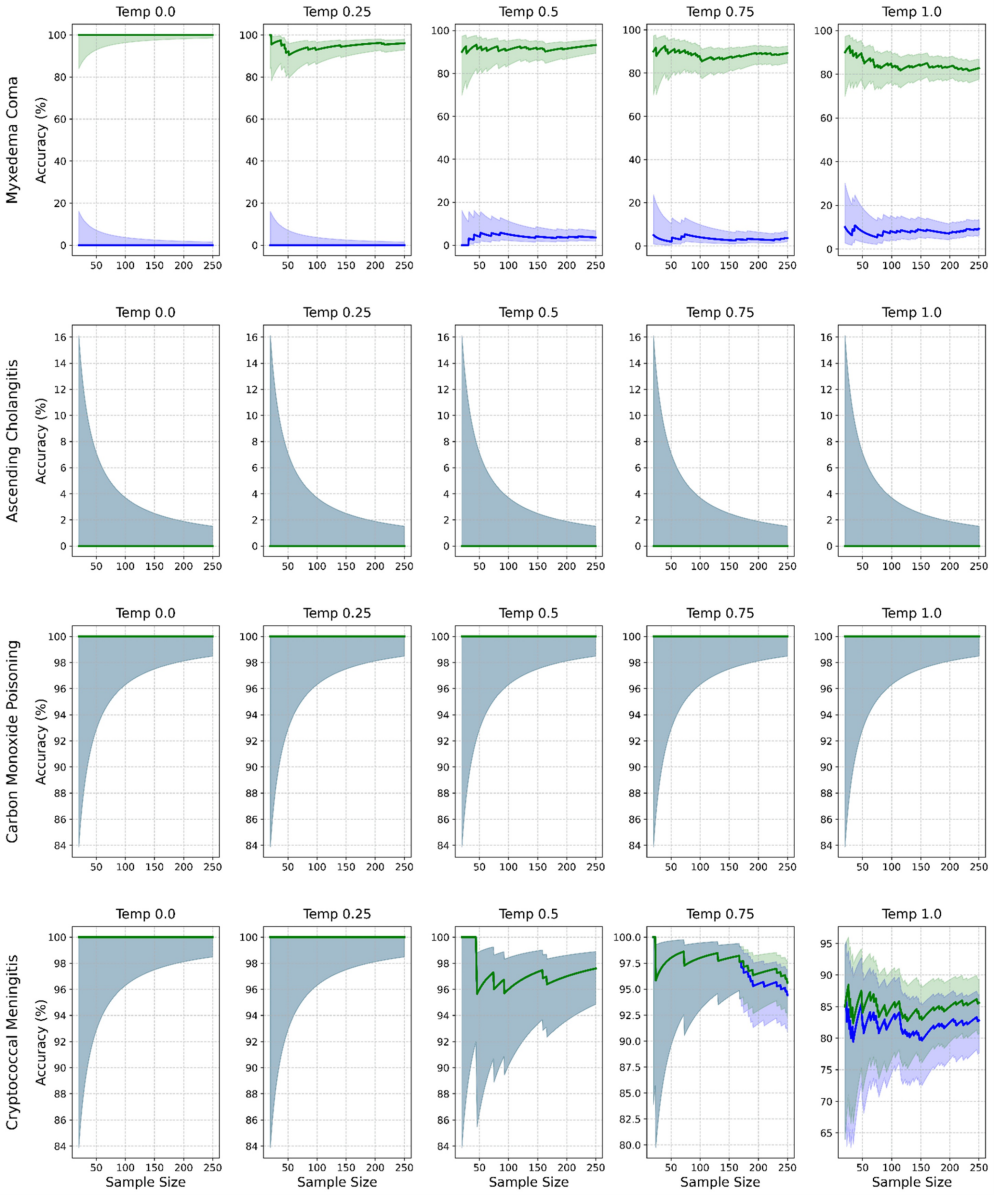
Diagnostic accuracy without physical examination data. Diagnostic accuracy by case (rows) and temperature (columns) without physical examination data. Lines show accuracy for leading (blue) and differential (green) diagnoses. Shaded purple and green areas represent the 95% confidence interval (CI) for the leading and differential diagnoses, respectively. Blue-gray areas represent overlap in 95% CI.

The primary outcomes for this study were specified a priori. Diagnostic accuracy refers to the correctness of the diagnoses proposed by GPT-4o when compared against a pre-specified, definitive diagnosis for each case, known as the “gold standard.” Diagnostic accuracy was assessed in two ways: leading diagnosis (whether the single diagnosis listed by GPT-4o as its first and most likely diagnosis matched the gold-standard diagnosis for that case) and differential diagnosis (whether the gold-standard diagnosis for that case appeared anywhere within the three-item list of differential diagnoses provided by GPT-4o). Diagnostic accuracy was plotted with iterative sampling to demonstrate the stochasticity of diagnostic accuracy. Figure 2 plots diagnostic accuracy when physical examinations were included, while Figure 3 plots diagnostic accuracy when physical examination data were excluded in a sensitivity analysis.

Diagnostic divergence was measured by the total number of unique diagnoses proposed by the LLM for each case, tallied at each temperature setting. This metric quantifies inconsistency in diagnostic outputs across iterations with identical inputs, representing variability in the differential diagnosis across case repetitions.

Diagnostic accuracy scoring

A keyword search was used to score diagnostic accuracy for each of the 10,000 outputs. For each case, specific keyword phrases representative of the correct diagnosis were predefined: myxedema coma (“myxedema”), ascending cholangitis (“cholangitis”), carbon monoxide poisoning (“carbon monoxide,” “co”), and cryptococcal meningitis (“cryptococcal meningitis,” “cryptococcal meningoencephalitis,” “cryptococcal encephalitis”).

The listed diagnoses (leading and three-item differential) provided by GPT-4o were searched for these keywords (case-insensitive). If a keyword was found in the leading diagnosis, it was scored as correct for “Leading Diagnosis.” If found in any of the three differential diagnoses, it was scored as correct for “Differential Diagnosis.”

To validate this keyword scoring method, two medical students (JH, LVO), blinded to the keyword scores and temperature settings, independently reviewed 1,000 outputs (250 from each case, sampled evenly across all temperatures and physical examination inclusion states). The reviewers scored the leading and differential diagnoses as correct or incorrect based on the gold-standard diagnosis for each case. Disagreements between the two student reviewers were adjudicated by a board-certified EM physician (PJ). Following adjudication, there was perfect concordance between the keyword search and human review (Cohen’s κ = 1.0), confirming the reliability of this systematic scoring method.

Mixed-effects modeling

We modeled diagnostic accuracy using mixed-effects logistic regression with temperature (0.0-1.0) as a continuous predictor across the four cases with physical examinations. Repeated case iterations were clustered to account for within-case correlation; inference used cluster-robust standard errors (primary) and, in sensitivity analyses, generalized estimating equations (GEEs) with exchangeable correlation at the case-temperature pair. Separate models evaluated (i) correct leading diagnosis and (ii) inclusion of the gold-standard diagnosis anywhere in the differential. We report β, odds ratios (ORs = exp(β)), 95% confidence intervals (CIs), and p-values. Intraclass correlation coefficients were estimated from GEE to quantify within-cell dependence.

Diagnostic divergence analysis

Each individual LLM output was constrained to a three-item differential diagnosis, representing the model’s top-priority conditions based on the presented case. However, when the same case is presented repeatedly, the model may generate different sets of diagnoses each time due to the non-deterministic nature of LLMs. This across-iteration variability, despite identical input, represents diagnostic inconsistency that may undermine reliability.

**Figure 4:**
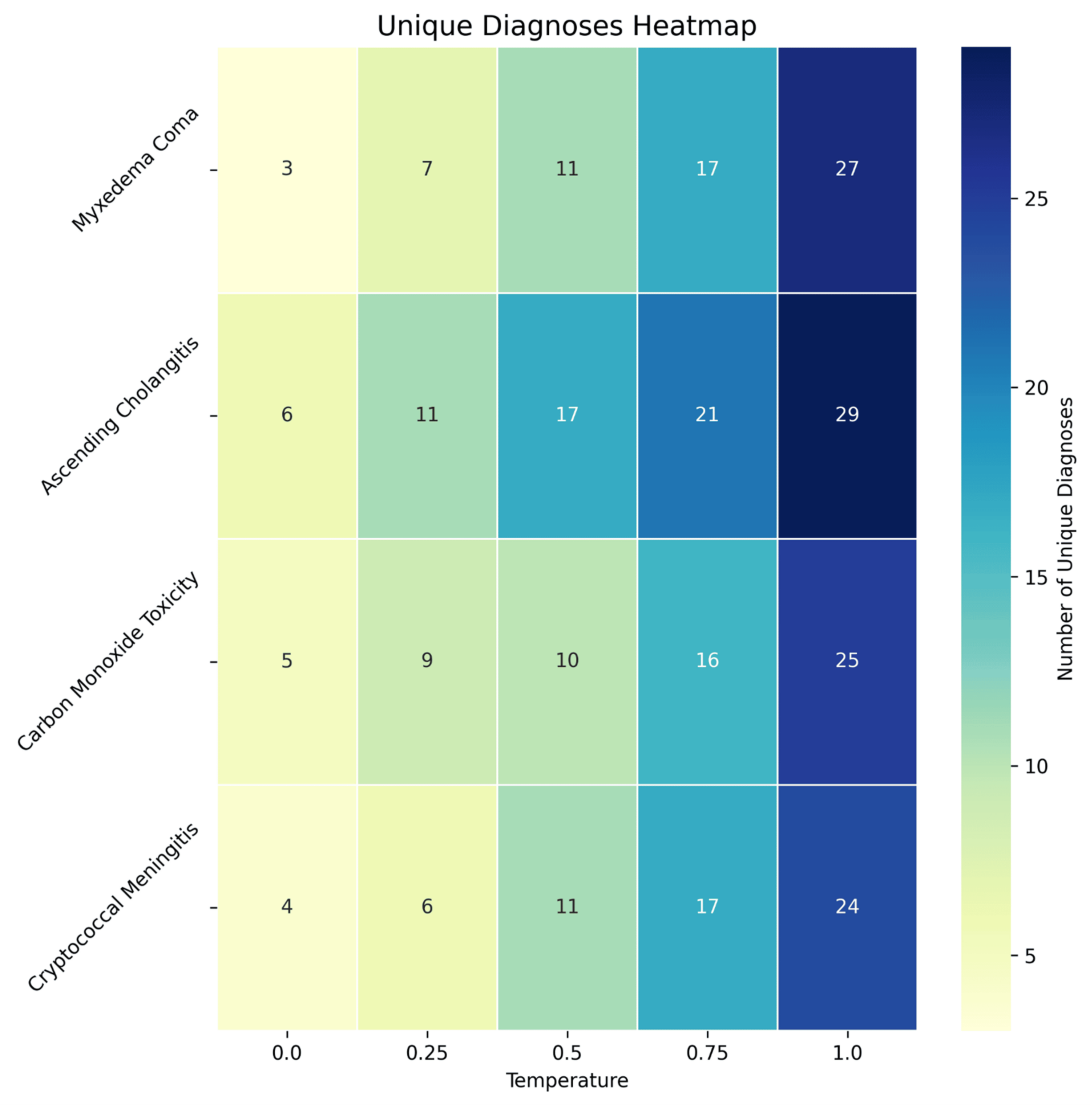
Diagnostic divergence by case. Heatmap depicting the number of standardized diagnoses considered in the three-item differential diagnoses across repetitive samples for each case-temperature pair. Diagnoses may overlap, such that items counted at low temperatures may also be counted at higher temperatures.

**Figure 5:**
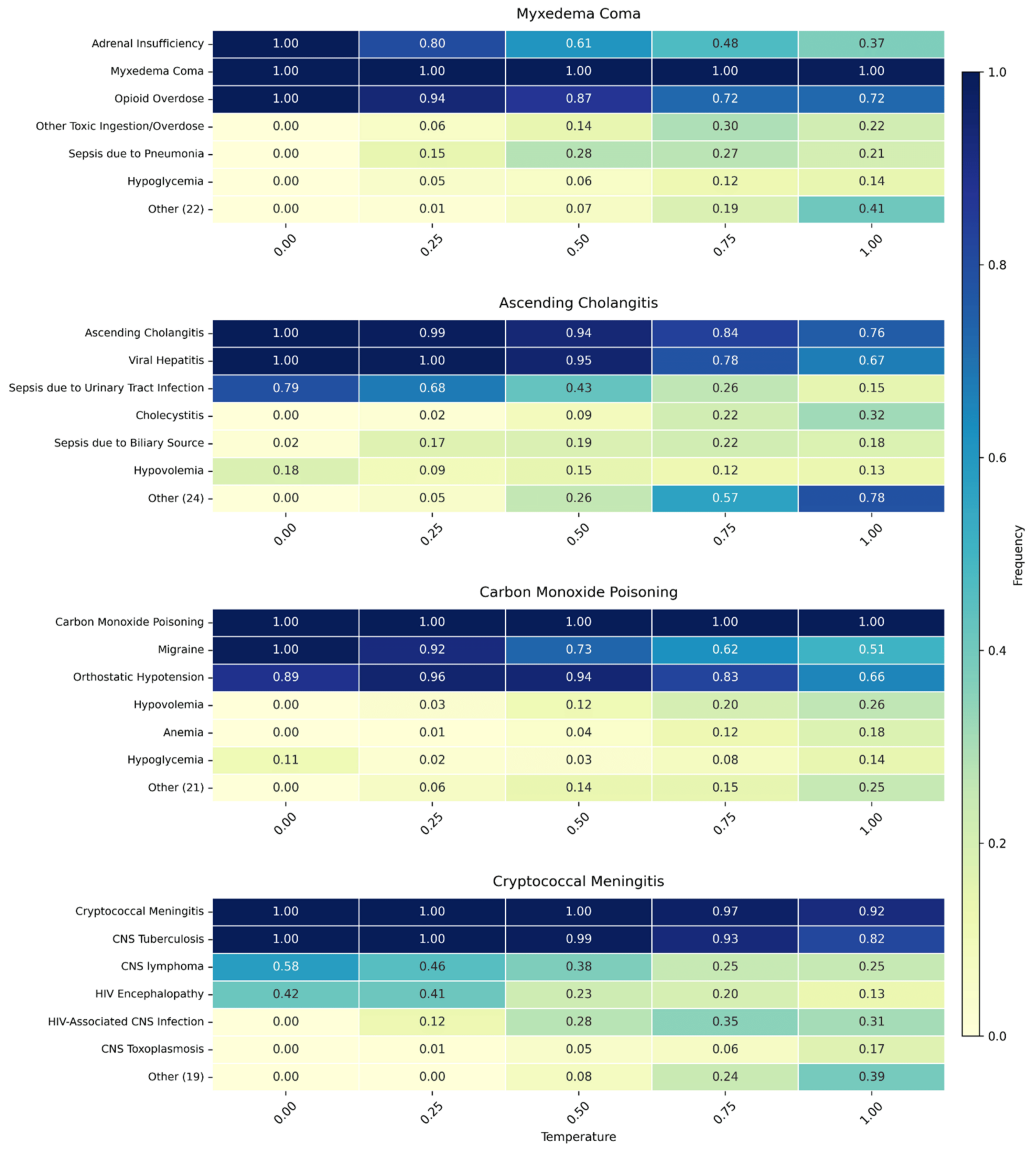
Unique diagnoses by case. Heatmap depicting the frequency of unique standardized diagnoses in the three-item differential at each case-temperature pair. The “Other” diagnosis represents the cumulative frequency of all additional diagnoses considered for each case.

To quantify this inconsistency, we measured diagnostic divergence as the total number of unique diagnoses generated across all iterations of a given case at each temperature setting (Figure 4). The relative frequencies of each diagnosis across case iterations are provided in Figure 5. To isolate the role of temperature in this analysis, only cases that included physical examination data were included (n = 5,000). Because diagnoses were often expressed with subtle differences in terminology, all outputs were standardized to group synonymous or conceptually equivalent terms. This allowed us to calculate the total number of distinct, standardized diagnostic categories per case-temperature pair. Synonymous labels (e.g., heart attack, myocardial infarction, acute coronary syndrome) were merged under a single canonical term to enable consistent comparison of diagnostic diversity across cases and temperature settings.

Statistical analysis

Diagnostic accuracy (leading and differential) was calculated as a percentage with corresponding 95% CIs using the Wilson score interval method. These metrics were calculated for each case-temperature pair. The number of unique diagnoses (diagnostic divergence) was tabulated. All analyses were performed using Python (version 3.9).

Ethics and reporting

This study was approved by the Institutional Review Board of the lead author’s academic institution during expedited review of a parent protocol. The present study does not involve human subjects. The authors adhered to the TRIPOD-LLM Reporting Guidelines for studies using or evaluating LLMs. This article was previously posted to the medRxiv preprint server on June 6, 2025.

## Results

Diagnostic scoring procedure

The keyword search was applied to all 10,000 LLM outputs to score leading and differential diagnoses as correct or incorrect. To validate this approach, a random 10% sample (n = 1,000) was independently reviewed by two medical students who were successfully blinded to the scores of the keyword search function. Reviewer agreement was high, with only three discrepancies between humans across all 1,000 cases (Cohen’s κ = 0.99). These three cases were escalated to a board-certified emergency physician, also blinded to the keyword-based scores. In all instances, the tiebreak aligned with the keyword search. Following adjudication, there was perfect concordance between the keyword search and human review (Cohen’s κ = 1.0), confirming the reliability of this systematic scoring method.

Diagnostic accuracy

When provided with physical examination data, GPT-4o demonstrated high diagnostic accuracy, particularly at lower temperature settings, achieving 100% accuracy for the leading diagnosis at temperature 0.0 across all cases. As the temperature increased, a clear trend of decreasing accuracy was observed (Table 3). Overall, leading diagnosis accuracy declined from 100% at temperature 0.0 to 89.4% at temperature 1.0 (95% CI = 85.8-91.8). Accuracy of the three-item differential diagnosis remained higher but showed a similar decline, dropping from 100% at lower temperatures to 92.6% at temperature 1.0 (95% CI = 89.3-94.6). The impact of temperature varied significantly by case (Table 3). Ascending cholangitis demonstrated the most sensitivity to temperature changes, with leading diagnosis accuracy dropping from 100% at temperature 0.0 to 70.4% at temperature 1.0 (95% CI = 64.5-75.7). In contrast, carbon monoxide poisoning maintained 100% accuracy across all temperatures, likely due to a pathognomonic presentation. Myxedema coma and cryptococcal meningitis showed intermediate sensitivity to temperature increases.

**Table 3:** Diagnostic accuracy by case-temperature pair. Diagnostic accuracy (95% confidence intervals (CIs)) of GPT-4o on each case at five pre-specified temperatures, including and excluding physical examination findings.

Case	Temperature	Accuracy with examinations (95% CI)		Accuracy without examinations (95% CI)	
		Leading	Differential	Leading	Differential
Myxedema coma	0.0	100% (98.5-100)	100% (98.5-100)	0% (0-1.5)	100% (98.5-100)
	0.25	100% (98.5-100)	100% (98.5-100)	0% (0-1.5)	96% (92.8-97.8)
	0.5	100% (98.5-100)	100% (98.5-100)	3.6% (1.9-6.7)	93.2% (89.4-95.7)
	0.75	99.6% (97.8-99.9)	99.6% (97.8-99.9)	3.6% (1.9-6.7)	89.2% (84.7-92.5)
	1.0	98.4% (96-99.4)	99.2% (97.1-99.8)	9.2% (6.2-13.4)	82.8% (77.6-87)
Ascending cholangitis	0.0	100% (98.5-100)	100% (98.5-100)	0% (0-1.5)	0% (0-1.5)
	0.25	98.8% (96.5-99.6)	98.8% (96.5-99.6)	0% (0-1.5)	0% (0-1.5)
	0.5	94% (90.3-96.3)	94% (90.3-96.3)	0% (0-1.5)	0% (0-1.5)
	0.75	81.6% (76.3-85.9)	85.2% (80.3-89.1)	0% (0-1.5)	0% (0-1.5)
	1.0	70.4% (64.5-75.7)	78.8% (73.3-83.4)	0% (0-1.5)	0% (0-1.5)
Carbon monoxide toxicity	0.0	100% (98.5-100)	100% (98.5-100)	100% (98.5-100)	100% (98.5-100)
	0.25	100% (98.5-100)	100% (98.5-100)	100% (98.5-100)	100% (98.5-100)
	0.5	100% (98.5-100)	100% (98.5-100)	100% (98.5-100)	100% (98.5-100)
	0.75	100% (98.5-100)	100% (98.5-100)	100% (98.5-100)	100% (98.5-100)
	1.0	100% (98.5-100)	100% (98.5-100)	100% (98.5-100)	100% (98.5-100)
Cryptococcal meningitis	0.0	100% (98.5-100)	100% (98.5-100)	100% (98.5-100)	100% (98.5-100)
	0.25	100% (98.5-100)	100% (98.5-100)	100% (98.5-100)	100% (98.5-100)
	0.5	99.6% (97.8-99.9)	99.6% (97.8-99.9)	97.6% (94.9-98.9)	97.6% (94.9-98.9)
	0.75	93.2% (89.4-95.7)	97.2% (94.3-98.6)	94.4% (90.8-96.6)	95.6% (92.3-97.5)
	1.0	88.8% (84.3-92.1)	92.4% (88.4-95.1)	82.8% (77.6-87)	85.6% (80.7-89.4)
Overall	All	96.2% (95.7-96.7)	97.2% (96.7-97.7)	49.6% (48.2-50.9)	72% (70.7-73.2)

Mixed-effects modeling

We fit mixed-effects logistic models across the four cases (with physical examinations) to quantify the association between sampling temperature (0-1) and diagnostic accuracy. Temperature was inversely associated with correct leading diagnosis (β = -4.16, OR = 0.02, 95% CI = 0.01-0.03, p < 0.001) and with inclusion of the gold-standard diagnosis anywhere in the differential (β = -3.75, OR = 0.02, 95% CI = 0.01-0.05, p < 0.001). Within-case correlation was addressed using clustered binomial models with robust standard errors by case. The intraclass correlation across the 250 iterations per case-temperature pair, estimated via GEE with an exchangeable working correlation, was 0.08 for leading accuracy and 0.06 for differential accuracy, indicating modest within-cell dependence. These estimates support a strong adverse association between higher sampling temperature and diagnostic accuracy when clinical examination findings are present.

Sensitivity analysis of physical examination

Excluding physical examination data led to a substantial reduction in diagnostic performance overall, with accuracy for the leading diagnosis dropping to 49.6% (95% CI = 48.2-51.0) and the three-item differential to 72.0% (95% CI = 70.7-73.2). The effect varied by case. Myxedema coma was particularly sensitive in terms of the leading diagnosis, which dropped to 3.3% (95% CI = 2.4-4.4), although the correct diagnosis still appeared within the differential list in 92.2% (95% CI = 90.6-93.6) of iterations. Ascending cholangitis showed complete dependence on physical examination findings, with diagnostic accuracy falling to 0% for both the leading and differential diagnoses in their absence. Carbon monoxide poisoning was unaffected by the removal of physical examination data, with diagnostic accuracy remaining at 100% across all temperatures. Cryptococcal meningitis showed only minimal sensitivity to physical examination data, with leading diagnosis accuracy at 95.0% (95% CI = 93.6-96.0) and differential diagnosis accuracy at 95.8% (95% CI = 94.5-96.7).

Diagnostic divergence analysis

To isolate the impact of temperature on diagnostic divergence, this analysis was limited to cases that included physical examination data. Across all case-temperature pairs, GPT-4o generated 371 unique raw diagnoses (case-insensitive). These were subsequently consolidated into 91 standardized diagnostic categories through human review.

Temperature had a significant effect on the inconsistency of diagnoses generated by GPT-4o across iterations (Figure 4). Increasing temperature consistently led to greater diagnostic divergence for each case. Myxedema coma showed unique diagnoses increasing from 3 at temperature 0.0 to 27 at temperature 1.0. Ascending cholangitis demonstrated unique diagnoses rising from 6 at temperature 0.0 to 29 at temperature 1.0. Carbon monoxide poisoning showed unique diagnoses increasing from 5 at temperature 0.0 to 25 at temperature 1.0. Cryptococcal meningitis exhibited unique diagnoses growing from 4 at temperature 0.0 to 24 at temperature 1.0.

Overall, the average number of unique diagnoses was 4.5 at temperature 0.0, increasing to an average of 26.25 at temperature 1.0. This represents a 483% increase in diagnostic divergence across iterations of the same case. Higher temperatures led to inconsistent three-item differentials across case iterations.

As the temperature increased to 1.0, the correct diagnosis often remained one of the most frequent, but its overall frequency typically decreased, and a larger set of alternative diagnoses appeared more often (Figure 5). For instance, in the case of ascending cholangitis at temperature 1.0, the correct diagnosis was still a common response, but other conditions appeared with increasing frequency, diluting diagnostic consensus across iterations.

## Discussion

This pilot study systematically evaluated the influence of the temperature parameter on GPT-4o’s diagnostic performance using four challenging EM vignettes with data inputs typical of an early patient encounter. Temperature significantly impacted accuracy, consistent with prior work cautioning that sampling temperature modulates randomness and can degrade task performance if set too high [16-18] and with vendor documentation noting non-determinism in model outputs [14]. Although repeated runs within the same case-temperature pair showed slight correlation in our mixed-effects model, intraclass correlation coefficients were low (0.06-0.08), suggesting clustering had only a modest effect and that the observed decline with higher temperatures is likely attributable to temperature itself [16-18].

The primary observation is the potential harm of temperature on diagnostic accuracy: lower temperatures yielded consistent, highly accurate outputs within and between case iterations, aligning with recommendations to use low temperature for focused, reproducible responses in clinical contexts [16-18]. At these settings, GPT-4o reliably identified the correct diagnosis as its leading choice, concordant with reports that LLMs can approach or match clinician-level diagnostic performance under well-controlled conditions [1-3,6].

Conversely, higher temperatures produced greater diagnostic divergence across iterations, degrading leading diagnosis accuracy and compromising reliability [16-18]. For ascending cholangitis, the decline was substantial. In settings where tools are queried once (e.g., synchronous CDS or consumer chatbots), such stochasticity may introduce unacceptable uncertainty, and consistent with concerns about verbose, sensitivity, maximizing differentials that can inflate apparent performance, does not necessarily translate into better diagnostic lists [22].

These results are relevant to ongoing applications of LLMs in healthcare. In ambient documentation systems that draft assessments and impressions, low temperature may support accurate, consistent labeling of routine conditions, whereas “turning up” temperature to force exploration risks noise unless carefully scaffolded [13,16]. For triage and diagnostic decision support in the ED, preference for deterministic settings accords with emerging evidence on LLM-enabled triage/diagnosis and prospective CDS evaluations in emergency care [7-12].

Temperature settings are often hidden in commercial products, and stochasticity reporting remains inconsistent, complicating appraisal of reliability and reproducibility [14-16,18]. Such opacity has implications for trust, governance, and medicolegal accountability in high-stakes environments where clinicians must act on model outputs quickly and confidently [14-16,18].

Practically, our findings suggest two implementation heuristics. First, default to low temperature for documentation and CDS tasks where precision and repeatability are paramount [16-18]. Second, when broader exploration is desired (e.g., hypothesis generation), prefer structured prompt strategies or deliberate multi-sample aggregation over simply increasing temperature, which primarily amplifies inconsistency [16-18].

Case-specific differences also emerged: carbon monoxide poisoning maintained 100% accuracy across all temperatures, likely reflecting pathognomonic cues in the vignette, whereas ascending cholangitis, with overlapping symptomatology, was temperature-sensitive. A similar pattern of sensitivity was observed with respect to the inclusion or exclusion of physical examination data. For cases in which pathognomonic features may be heavily represented in the HPI, diagnostic accuracy was preserved in the absence of the physical examination data. However, ascending cholangitis and myxedema coma possess characteristic physical examination findings that, if excluded, could compromise diagnostic accuracy. This heterogeneity echoes task- and case-mix dependence seen in prior LLM diagnostic studies, including EM settings [1-3,6,7,11].

Finally, methodological transparency is essential. Temperature materially influenced performance here, yet it is underreported in LLM clinical studies. Consistent disclosure of temperature, top-p, context windows, and other decoding settings should be standard, aligned with calls for better reporting and with community guidance for LLM evaluations in medicine [15,19]. As LLMs enter safety-critical workflows, temperature tuning should be matched to use case (triage vs. collaborative reasoning vs. research), and human oversight should remain central, consistent with controlled trials and multi-site evaluations of AI-supported diagnostic processes [1-3,11,12].

Limitations

We prioritized sampling across case iterations (250 iterations per case-temperature pair) rather than case variety, resulting in the inclusion of only four EM vignettes. Importantly, simulated cases cannot replicate the ambiguity of real-world patient presentations, limiting representativeness. Scoring also relied on discrete gold‑standard diagnoses rather than probabilistic reasoning. We restricted LLM diagnostic outputs to a three-item differential diagnosis, which may favor precision. Only one LLM model/version was tested; generalizability to other models and future releases is unknown. OpenAI models did not permit specification of the random seed, which could confound the stochasticity across case iterations when the temperature is greater than 0.0. Finally, the impact of temperature on reasoning models was not tested.

## Conclusions

In this pilot study, temperature was a major determinant of diagnostic performance across four EM vignettes. Low temperature optimized accuracy and reproducibility, while high settings amplified diagnostic divergence. Transparent reporting and thoughtful tuning are essential as LLMs enter safety‑critical workflows such as EM.
